# Modulation of Second Messenger Signaling in the Brain Through PDE4 and PDE5 Inhibition: Therapeutic Implications for Neurological Disorders

**DOI:** 10.3390/cells14020086

**Published:** 2025-01-09

**Authors:** Min Kyu Park, Hyun Wook Yang, Seo Young Woo, Dong Yeon Kim, Dae-Soon Son, Bo Young Choi, Sang Won Suh

**Affiliations:** 1 Department of Physiology, College of Medicine, Hallym University, Chuncheon 24252, Republic of Korea; bagmingyu50@gmail.com (M.K.P.); akqjqtj5@hallym.ac.kr (H.W.Y.); 1wsy@naver.com (S.Y.W.); roy8596@naver.com (D.Y.K.); 2 Division of Data Science, Data Science Convergence Research Center, Hallym University, Chuncheon 24252, Republic of Korea; biostat@hallym.ac.kr; 3 Institute of Sport Science, Hallym University, Chuncheon 24252, Republic of Korea; bychoi@hallym.ac.kr; 4 Department of Physical Education, Hallym University, Chuncheon 24252, Republic of Korea

**Keywords:** phosphodiesterase (PDE), phosphodiesterase 4 (PDE4), phosphodiesterase 5 (PDE5), protein kinase A (PKA), protein kinase G (PKG)

## Abstract

Phosphodiesterase (PDE) enzymes regulate intracellular signaling pathways crucial for brain development and the pathophysiology of neurological disorders. Among the 11 PDE subtypes, PDE4 and PDE5 are particularly significant due to their regulation of cyclic adenosine monophosphate (cAMP) and cyclic guanosine monophosphate (cGMP) signaling, respectively, which are vital for learning, memory, and neuroprotection. This review synthesizes current evidence on the roles of PDE4 and PDE5 in neurological health and disease, focusing on their regulation of second messenger pathways and their implications for brain function. Elevated PDE4 activity impairs synaptic plasticity by reducing cAMP levels and protein kinase A (PKA) activity, contributing to cognitive decline, acute brain injuries, and neuropsychiatric conditions such as bipolar disorder and schizophrenia. Similarly, PDE5 dysregulation disrupts nitric oxide (NO) signaling and protein kinase G (PKG) pathways, which are involved in cerebrovascular homeostasis, recovery after ischemic events, and neurodegenerative processes in Alzheimer’s, Parkinson’s, and Huntington’s diseases. PDE4 and PDE5 are promising therapeutic targets for neurological disorders. Pharmacological modulation of these enzymes offers potential to enhance cognitive function and mitigate pathological mechanisms underlying brain injuries, neurodegenerative diseases, and psychiatric disorders. Further research into the regulation of PDE4 and PDE5 will advance therapeutic strategies for these conditions.

## 1. Introduction

Phosphodiesterases (PDEs) are enzymes essential for cellular signaling, responsible for breaking down cyclic nucleotides like cyclic adenosine monophosphate (cAMP) and cyclic guanosine monophosphate (cGMP) [1]. These cyclic nucleotides act as key second messengers in numerous physiological processes, including metabolism, vascular regulation, and neuronal signaling [1]. The PDE family is diverse, consisting of 11 subfamilies (PDE1–PDE11), each with distinct tissue distribution, substrate specificity, and regulatory mechanisms, reflecting their specialized roles in various biological systems [1].

Among the PDE family, PDE4 and PDE5 have garnered particular interest due to their involvement in brain function and neurological disorders [2,3]. PDE4 primarily regulates cAMP levels in neurons, influencing intracellular signaling pathways that govern synaptic plasticity, neuronal survival, and memory formation [4,5,6,7].

Dysregulation of PDE4 and PDE5 activity has been implicated in the pathology of several neurodegenerative and neuropsychiatric disorders, including Alzheimer’s disease, stroke, and cognitive impairments [3,8,9,10,11,12]. As a result, pharmacological inhibitors targeting these enzymes have emerged as promising therapeutic strategies. PDE4 inhibitors enhance cAMP signaling, promoting neuroprotection and cognitive improvements, while PDE5 inhibitors restore cGMP signaling, supporting cerebral blood flow, reducing oxidative stress, and mitigating neuronal damage [3,8,9,10,11,12].

This review focuses on the roles of PDE4 and PDE5 in neurological health and disease, highlighting their mechanisms of action, therapeutic potential, and the latest advancements in pharmacological modulation.

## 2. Distribution and Functional Roles of PDE4 and PDE5 Isoforms in the Central Nervous System and Peripheral Tissues

### 2.1. PDE4 Isoforms in the CNS and Peripheral Tissues

**Figure 1:**
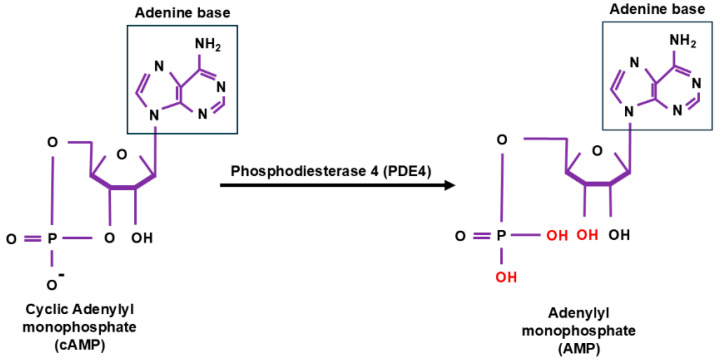
The chemical structures of cyclic adenosine monophosphate (cAMP) and adenosine monophosphate (AMP) [20]. Phosphodiesterase type 4 (PDE4) catalyzes the hydrolysis of cAMP into AMP [20]. In cAMP, the 3′ and 5′ hydroxyl groups of the adenosine ribose are connected by a phosphodiester bond, forming its characteristic cyclic structure [20]. PDE4 cleaves the phosphodiester bond at the 3′ position, disrupting the cyclic structure and resulting in the formation of a linear AMP molecule [20]. This hydrolysis reaction involves the addition of water, yielding AMP and inorganic phosphate (Pi) as final products [20].

The PDE4 family includes four isoforms PDE4A, PDE4B, PDE4C, and PDE4D each exhibiting distinct expression patterns and physiological roles [13,14]. In the CNS, PDE4 isoforms are broadly expressed in neurons and glial cells and are notably persistent in aged and Alzheimer’s disease-affected brains, suggesting potential roles in neurodegeneration [15,16]. Among these, PDE4B is the most widely distributed, with high expression in brain regions such as the cortex, hippocampus, and cerebellum [17,18,19]. PDE4A is also abundant in the cortex but is expressed at two to four times lower levels in other areas [17]. PDE4D is prominent in the frontal cortex but is generally less abundant than PDE4B in most CNS regions. Conversely, PDE4C is minimally expressed in the brain, indicating a limited or specialized role [17] (Figure 1). 

In peripheral tissues, PDE4B and PDE4D are predominant, contributing to immune regulation, inflammation, and vascular function. Dysregulation of PDE4 activity has been associated with psychiatric conditions, including depression and schizophrenia, due to its influence on cAMP signaling pathways [17,18,19]. PDE4 also regulates ion channels involved in neuronal excitability, underscoring its importance in maintaining neural communication and preventing hyperactivity [17,18,19]. 

### 2.2. PDE5 Isoforms in the CNS and Peripheral Tissues

**Figure 2:**
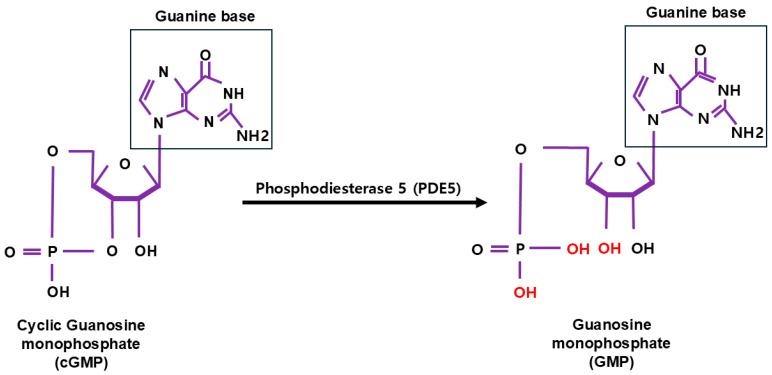
The chemical structures of cyclic guanosine monophosphate (cGMP) and guanosine monophosphate (GMP) [24]. Phosphodiesterase type 5 (PDE5) catalyzes the hydrolysis of cGMP into GMP. In cGMP, a phosphodiester bond links the 3′ and 5′ hydroxyl groups of the guanosine ribose, creating its cyclic structure [24]. In cGMP, a phosphodiester bond links the 3′ and 5′ hydroxyl groups of the guanosine ribose, forming a cyclic structure [24]. PDE5 cleaves this bond at the 3′ position, breaking the cyclic structure and converting cGMP into its linear form, GMP [24]. This hydrolysis reaction involves the addition of water, producing GMP and inorganic phosphate (Pi) as final products [24].

Unlike PDE4, PDE5 primarily exists as a single isoform. Its expression is robust in peripheral tissues, including vascular smooth muscle, platelets, and the gastrointestinal tract, where it regulates vascular tone, platelet aggregation, and smooth muscle contraction [3,21,22,23]. In the CNS, PDE5 expression is relatively limited but regionally specific [3,21,22,23]. It is prominently expressed in the hippocampus, cerebellum, and cortex, regions critical for cognitive functions and synaptic plasticity [3,21,22,23]. In the hippocampus, PDE5 is involved in processes such as memory formation and long-term potentiation (LTP), mediated by the cGMP-dependent nitric oxide (NO)-cGMP signaling pathway [3,21,22,23]. Moderate expression in the cerebellum suggests a role in motor coordination, while cortical distribution varies based on localized cGMP signaling demands [3,21,22,23] (Figure 2). 

### 2.3. Integration of Roles in Signaling and Pathophysiology

PDE4 and PDE5 regulate complementary but distinct pathways, with PDE4 focusing on cAMP and PDE5 on cGMP. Both enzymes ensure the balance of second messengers critical for neuronal signaling and plasticity [25,26]. PDE4 activity governs PKA-mediated phosphorylation of targets like CREB, influencing gene expression essential for learning, memory, and neuronal survival [25,26]. Similarly, PDE5 modulates PKG activation through NO-cGMP signaling, affecting synaptic transmission and neurovascular coupling [25,26].

The dysregulation of these pathways has significant pathological implications. In the CNS, altered PDE4 activity has been linked to neuropsychiatric disorders, while PDE5 dysfunction is implicated in neurodegenerative conditions such as Alzheimer’s disease and cerebrovascular disorders like stroke [3,27,28]. Notably, PDE5 regulates cerebral blood flow by modulating vascular tone, playing a crucial role in brain homeostasis and protection against ischemic injury [28].

### 2.4. Therapeutic Implications

The distinct and region-specific roles of PDE4 and PDE5 in the CNS present unique opportunities for therapeutic targeting. PDE4B and PDE4D, due to their prominence in synaptic function, may be viable targets for treating psychiatric and neurodegenerative disorders [25,26,27,28]. Similarly, PDE5’s targeted expression in cognitive-related brain regions highlights its potential for interventions aimed at memory enhancement and neuroprotection. By selectively modulating these isoforms, therapies can address disorders linked to dysregulated cAMP or cGMP signaling while minimizing off-target effects [25,26,27,28].

## 3. Therapeutic Potential of PDE4 and PDE5 in Neurological Disorders

Among the diverse phosphodiesterase subtypes, PDE4 and PDE5 have garnered significant attention due to their crucial involvement in the pathophysiology of several brain disorders. Inhibition of these enzymes shows promising therapeutic potential, particularly in managing traumatic brain injury (TBI), ischemic stroke, and epilepsy [1,26]. By modulating intracellular cAMP and cGMP levels, targeted interventions involving PDE4 and PDE5 may provide therapeutic advantages for both acute and progressive neurological conditions [29].

**Figure 3:**
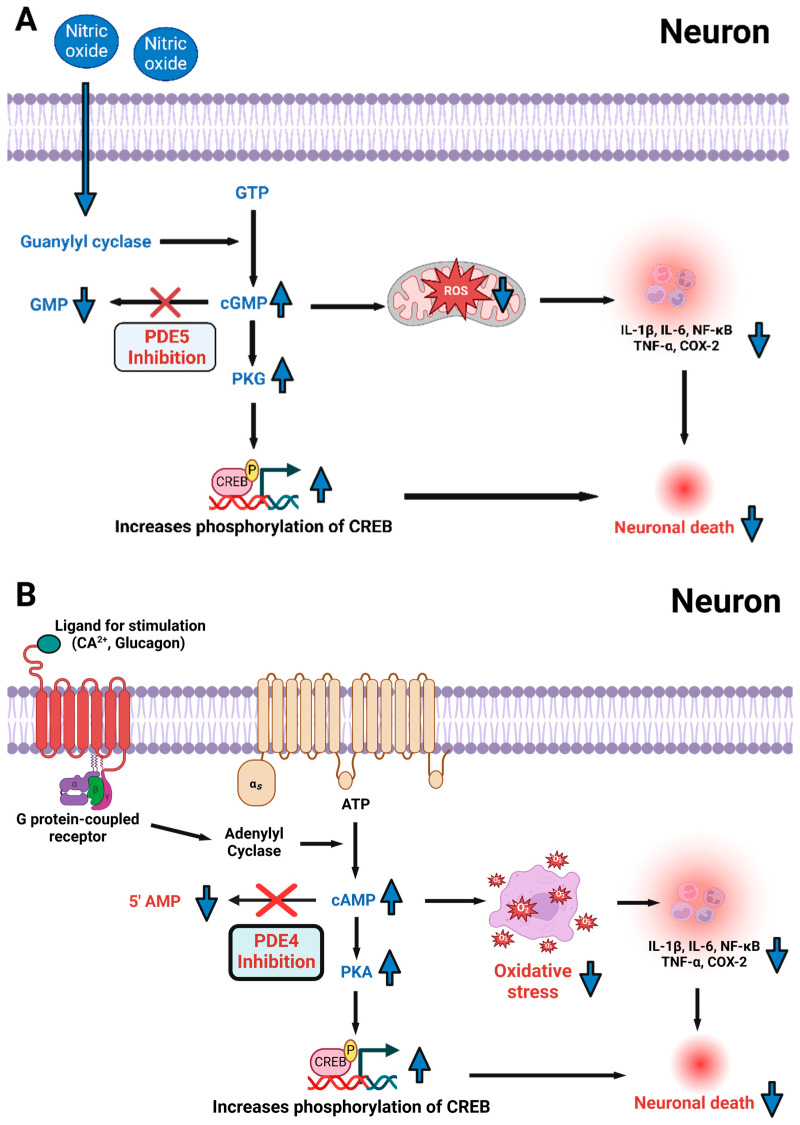
The diagram highlights the roles of PDE4 and PDE5 in regulating second messenger systems and their therapeutic relevance in neurological disorders [16,23]. (**A**) PDE4 controls cAMP levels, with its inhibition activating PKA and phosphorylating CREB, promoting neuronal survival, synaptic plasticity, cognitive function, and anti-inflammatory responses [9]. Elevated cAMP also reduces reactive oxygen species (ROS), alleviating oxidative stress [28]. (**B**) PDE5 inhibition prevents cGMP degradation, activating PKG, which enhances vasodilation, neuroprotection, and antioxidant defenses [34,35]. PKG also mitigates ROS production, reducing oxidative damage [34,35]. In traumatic brain injury (TBI), PDE4 and PDE5 inhibitors improve neuronal repair, reduce inflammation, and protect against oxidative stress [18,36,37]. In ischemic stroke, these inhibitors lower ROS, support neurogenesis, and facilitate recovery through CREB and PKG pathways [18,36,37,38]. In epilepsy, PDE4 inhibition regulates synaptic plasticity, while PDE5 inhibition stabilizes neuronal function by improving blood flow and reducing excitotoxicity [34,35]. In Alzheimer’s disease, combined PDE4 and PDE5 inhibition improves cognition, reduces amyloid-beta accumulation, and protects against oxidative stress [18,36,37,38]. (**A**,**B**) Together, PDE4 and PDE5 inhibitors strengthen the cAMP/CREB and cGMP/PKG pathways, offering broad neuroprotective benefits, reducing inflammation, and enhancing cognitive and synaptic function across multiple neurological conditions [2,3,9,28,39,40]. “Created with BioRender.com”.

PKA and PKG pathways, activated by cAMP and cGMP respectively, play essential roles in cellular processes associated with brain repair and neuroprotection [30]. PKA activity contributes to synaptic repair, reduces neuronal apoptosis, attenuates neuroinflammation, and helps preserve the integrity of the blood-brain barrier, all of which are essential for recovery following brain injury [31,32]. In parallel, PKG signaling mitigates oxidative stress and combats mitochondrial dysfunction, key pathological features observed in TBI and other neurodegenerative conditions [33]. This dual modulation of PKA and PKG pathways offers a promising framework for developing therapeutic strategies targeting PDE4 and PDE5 to mitigate brain injury, restore neural function, and protect against future degeneration (Figure 3).

### 3.1. Seizure and Epilepsy

**Figure 4:**
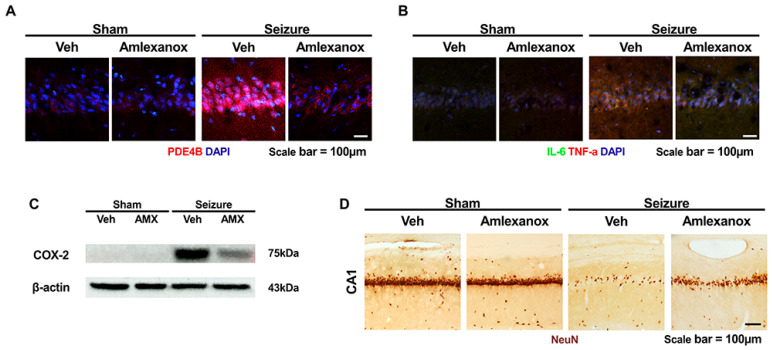
One week after epilepsy induction, brains were harvested to evaluate the effects of the PDE4 inhibitor amlexanox. NeuN staining demonstrates preserved neuronal populations in the CA1 region of the hippocampus in the amlexanox-treated group, indicating a neuroprotective effect [26]. (**A**) Immunofluorescence images show overexpression of PDE4B within neurons in the hippocampal CA1 region 1 week after epilepsy, detected using PDE4B immunohistochemistry with specific antibodies PDE4B [26]. PDE4 staining indirectly verifies the efficacy of the PDE4 inhibitor by detecting a decrease in the intensity of PDE4 expression (PDE4B, red) [26]. (**B**) Immunofluorescence images show overexpression of PDE4B within neurons in the hippocampal CA1 region 1 week after epilepsy, detected using IL-6 and TNF-α immunohistochemistry with specific antibodies IL-6, TNF-α [26]. IL-6 and TNF-α staining indirectly verifies the efficacy of the PDE4 inhibitor by detecting a decrease in the intensity of inflammation expression (TNF-α, red) (IL-6, green) [26]. (**C**) COX-2, the final inflammatory marker, was confirmed to have a decreased protein expression level by PDE4 inhibitors through Western blot [26]. (**D**) Immunofluorescence images show live neurons in the hippocampal CA1 region 1 week after epilepsy, detected using NeuN [26]. It was confirmed that more neurons survived when PDE4 inhibitors were administered after epilepsy was induced [26]. COX-2 Western blot, along with TNF-α and IL-6 immunofluorescence, reveals decreased levels of pro-inflammatory markers, suggesting anti-inflammatory effects [26]. These findings underscore the potential of amlexanox to mitigate neuronal damage and inflammation in epilepsy [26].

Seizures result from abnormal electrical activity in the brain, manifesting as a spectrum of neurological symptoms ranging from brief lapses in attention to severe convulsions [41]. Epilepsy, a chronic condition characterized by recurrent seizures, significantly affects quality of life [42]. The PKA and PKG pathways, which regulate neuronal excitability and synaptic function, play pivotal roles in the development and control of seizure activity and epilepsy [34,43,44,45,46] (Figure 4).

Several phosphodiesterases (PDEs), including PDE1, PDE2, PDE3, and particularly PDE4, contribute to cAMP degradation within neurons [47,48]. PDE4 is highly expressed in the central nervous system (CNS) and is crucial for maintaining appropriate cAMP levels, preventing excessive neuronal excitability driven by increased PKA activation [43,47,48]. Dysregulated cAMP signaling can lead to abnormal neuronal firing, contributing to the onset and persistence of seizures. PDE4 inhibitors reduce cAMP breakdown, modulating PKA activity to stabilize synaptic function and potentially prevent seizure progression [43,44]. These inhibitors have also demonstrated protective effects in models of pilocarpine-induced seizures, reducing neuronal cell death, oxidative stress, and inflammation while promoting lysosomal function and autophagy [26,43,44]. The PKG pathway, regulated by cGMP, offers neuroprotective benefits through the modulation of nitric oxide (NO) signaling, reduction of oxidative stress, and prevention of mitochondrial dysfunction, all of which are associated with seizures [26,43,44]. PKG activation also influences ion channel function, stabilizing neuronal excitability [34,35,45,46]. PDE5 inhibitors, which enhance PKG activity, have shown potential to reduce seizure frequency and severity by improving neuronal resilience and mitigating oxidative damage [34,45,46].

### 3.2. Ischemia and Stroke

Ischemia, particularly in the context of stroke, results from reduced cerebral blood flow and oxygen supply, causing widespread neuronal damage [28,49]. The PKA and PKG pathways play essential roles in the brain’s response to ischemia, promoting neuronal survival and supporting recovery processes [28,49,50].

During ischemic events, activation of the PKA pathway facilitates neural repair by enhancing synaptic plasticity, crucial for the restoration of damaged networks [51,52]. PKA also reduces neuronal apoptosis and neuroinflammation, helping to maintain the integrity of the blood-brain barrier and preventing further damage from inflammatory infiltration [51,52]. PDE4 inhibitors, by elevating cAMP levels and activating PKA, offer neuroprotective effects by promoting these recovery processes and improving outcomes in stroke [2]. Additionally, PKA activation triggers the Nrf-2/HO-1 pathway, reducing oxidative stress and improving cell viability, which highlights its therapeutic potential in both ischemia and neurodegenerative diseases [53]. Increased AMP, SIRT1, and phosphorylated AMPK have also been linked to protection against brain edema and blood-brain barrier dysfunction, further supporting PKA’s protective role [2].

Similarly, the PKG pathway, activated by cGMP, mitigates oxidative stress and mitochondrial dysfunction—two key contributors to neuronal death in stroke [27,50]. PKG enhances the expression of antioxidant enzymes and supports mitochondrial health, ensuring neurons maintain energy production and resist apoptosis [27,50]. Furthermore, PKG modulates cerebral blood flow and reduces inflammation, preserving the viability of ischemic brain tissue [27,50]. Although PDE4 inhibitors generally have fewer side effects, such as emesis, higher doses needed for antidepressant effects may increase the risk of adverse reactions, necessitating further research into optimal dosing strategies [27,50]. PDE5 inhibitors, which prevent cGMP degradation, boost PKG activity and offer neuroprotection by enhancing mitochondrial function and reducing reactive oxygen species (ROS) [27]. By stimulating both the PKG and PI3K/Akt pathways, PDE5 inhibitors present a synergistic therapeutic strategy to protect neurons, minimize cell death, and improve recovery outcomes in stroke [28].

### 3.3. Traumatic Brain Injury (TBI)

Following traumatic brain injury (TBI), elevated expression of PDE4B2 and PDE4D2 in the hippocampus leads to reduced cAMP levels, impacting neuronal and immune cell function [18,36]. Inhibiting PDE4 has been shown to restore long-term potentiation (LTP) in the hippocampus and improve basal synaptic transmission by modulating AMPA receptor transport [18,36]. PDE4D, which is predominantly expressed in microglia and immune cells, suggests that PDE4 inhibition also helps regulate the inflammatory response following TBI. Collectively, PDE4 inhibitors hold potential to enhance cognitive and synaptic function post-TBI by promoting synaptic plasticity and reducing inflammation [18,36].

PDE5 inhibitors offer additional neuroprotection in TBI by targeting the NO-cGMP pathway, mitigating vasospasm, and preventing ischemia—two key contributors to poor outcomes in events such as subarachnoid hemorrhage (SAH) [37,38]. Research shows that PDE5 inhibition reduces neuronal death following SAH without increasing intracranial pressure, confirming its safety and efficacy [37,38]. These inhibitors also improve cerebral blood flow regulation in injured brain tissue without elevating intracranial pressure, even under conditions of reduced mean arterial pressure, which is essential for preserving brain function and preventing further damage [37,38]. Furthermore, PDE5 inhibition reduces levels of endothelin-1, a potent vasoconstrictor implicated in early brain injury, thus enhancing cerebral perfusion during the acute phase of TBI [37,38]. By improving blood flow to damaged areas, PDE5 inhibitors limit ischemic injury, reduce neuronal death, and support recovery. As such, PDE5 inhibition presents a promising therapeutic strategy for TBI management by maintaining vascular function and protecting neurons from ischemic damage and cell death [37,38].

### 3.4. Alzheimer’s Disease

PDE4 inhibitors hold promise for treating Alzheimer’s disease (AD) with comorbid depression by targeting the PDE4B and PDE4D subtypes, which regulate mood and memory, respectively [9]. By inhibiting PDE4, these compounds elevate cAMP levels, activating the cAMP/CREB/BDNF signaling pathway, which supports neuroprotection, anti-apoptotic effects, and cognitive enhancement [9,39]. Studies in APP/PS1 transgenic mice—a model for AD—have demonstrated that PDE4 inhibitors improve cognitive function, reduce depressive behaviors, and decrease neuronal apoptosis by increasing the Bcl-2/Bax ratio [9,39]. Additionally, PDE4 inhibition restores cAMP and pCREB levels, which are associated with cognitive improvements [9,39].

PDE5 inhibitors also exhibit potential in AD treatment through multiple mechanisms. They reduce amyloid-beta (Aβ) and phosphorylated tau levels, enhancing cognitive function in animal models of AD [3,40]. PDE5 inhibition activates the cGMP/PKG/CREB pathway, increasing neurotrophic factors such as BDNF and NGF, preserving mitochondrial function, and preventing apoptosis [3,40]. These inhibitors also counteract Aβ-induced dysregulation by modulating glucocorticoid receptor activity and restoring Wnt/β-catenin signaling, promoting neuroprotection [3,40]. Furthermore, PDE5 inhibitors facilitate Aβ clearance by activating the autophagy-lysosome and ubiquitin-proteasome systems, reducing toxic protein accumulation [3]. Their impact on reducing intracellular calcium levels in pericytes may further enhance cerebral blood flow and improve vascular function [3,40].

### 3.5. Summary of Effects of PDE4 and PDE5 Inhibitors in Neurological Disorders

The regulation of PDE4 and PDE5 highlights the critical role of second messenger systems in brain health, making them promising targets for neurological disorders. Modulating these pathways offers therapeutic potential across a range of conditions, including traumatic brain injury, ischemic stroke, epilepsy, and neurodegenerative diseases (Table 1 and Table 2). Further research on PDE inhibitors may pave the way for innovative strategies to treat these debilitating conditions by enhancing cognitive function, preventing neurodegeneration, and promoting recovery [3,9,39,40].

This table highlights the therapeutic potential of various PDE4 inhibitors across a range of neurological disorders, as demonstrated in preclinical animal models. Amlexanox, in a seizure and epilepsy model using Sprague Dawley rats, showed neuroprotective effects, including reduced neuronal cell death, decreased neuroinflammation, and oxidative stress, as well as enhanced lysosomal function and autophagy [26]. Rolipram and FCPR03, in ischemia and stroke models using adult male C57BL/6 mice and Sprague Dawley rats, reduced neuronal cell death, neuroinflammation, and reactive oxygen species (ROS) production while preserving blood-brain barrier integrity and promoting SIRT1, p-AMPK, and AKT/GSK3β/β-catenin signaling pathways [2,54]. A33 and Rolipram, in traumatic brain injury (TBI) models with Sprague Dawley rats, demonstrated significant neuroprotective effects, including reduced neuronal death, enhanced cognitive and synaptic function, and increased synaptic plasticity while mitigating neuroinflammation [18,36]. Roflumilast, in an Alzheimer’s disease (AD) model using APP/PS1 double transgenic mice, showed reduced neuronal death, increased cAMP/CREB/BDNF signaling, and improved Bcl-2/Bax ratios [9]. These effects contributed to enhanced cognitive function and alleviation of depressive-like behavior. These results underscore the broad neuroprotective and therapeutic potential of PDE4 inhibitors in addressing key pathological processes in neurological disorders.

This figure illustrates the therapeutic potential of PDE5 inhibitors across various neurological disorders, as evidenced in preclinical animal studies. Sildenafil, in seizure and epilepsy models using Male Swiss mice and Wistar rats, exhibited neuroprotective effects via the NO-cGMP-PKG pathway, reducing ROS production and neuronal excitability [34,35]. In ischemia and stroke models using 10-day-old male Long–Evans rats, Sildenafil significantly reduced neuronal death, enhanced neuroprotection through the PKG pathway, decreased ROS production, and activated the PI3K/Akt/mTOR pathway while improving mitochondrial function [28]. In traumatic brain injury (TBI) models with twenty-eight Wistar-derived albino strain female rats, Sildenafil reduced neuronal death, improved cerebral blood flow without increasing intracranial pressure, decreased endothelin-1 levels, and enhanced cerebral perfusion [37]. Mirodenafil, in an Alzheimer’s disease (AD) model using APP-C105 AD mice, decreased amyloid-beta (Aβ) and tau levels, improved cognitive function, facilitated Aβ clearance through the autophagy-lysosome pathway, enhanced cGMP/PKG/CREB signaling, and supported mitochondrial function [3]. These findings underscore the diverse and significant neuroprotective benefits of PDE5 inhibitors, highlighting their potential as therapeutic agents in neurological disorders.

## 4. Discussion

Phosphodiesterase (PDE) enzymes play an essential role in hydrolyzing phosphodiester bonds, contributing to brain development and the pathogenesis of neurological diseases [1]. Among the 11 PDE subtypes, PDE4 and PDE5 are particularly significant for brain function due to their roles in regulating cAMP and cGMP signaling pathways [1,55] (Table 3).

Phosphodiesterase (PDE) enzymes play a crucial role in hydrolyzing phosphodiester bonds, influencing brain development and the pathogenesis of neurological diseases [1]. Among the 11 PDE subtypes, PDE4 and PDE5 are particularly important for brain function due to their regulation of cAMP and cGMP signaling pathways [1]. PDE4 specifically hydrolyzes cAMP, a second messenger critical for cognitive functions [56]. Increased PDE4 activity lowers cAMP levels, impairing PKA activity and disrupting synaptic plasticity, which is essential for memory and learning [26,56]. Proper regulation of PDE4 is necessary to prevent cognitive disorders such as Alzheimer’s disease and epilepsy [9]. Similarly, PDE5 controls cGMP degradation, and its dysregulation affects vascular function and neuronal survival, contributing to neurological diseases [1,50].

PDE4 inhibitors offer therapeutic benefits by stabilizing cAMP levels, enhancing PKA activity, and improving synaptic plasticity [9,26]. They also reduce neuroinflammation and oxidative stress, which may mitigate seizure severity and slow progression of epilepsy. PDE5 inhibitors enhance the cGMP-PKG pathway, modulate nitric oxide (NO) signaling, and reduce oxidative stress, showing potential in decreasing seizure frequency and severity [34,35].

Both PDE4 and PDE5 inhibitors contribute to neuroprotection during ischemic strokes. PDE4 inhibitors increase cAMP levels, activating PKA to reduce neuronal apoptosis, neuroinflammation, and oxidative stress, while maintaining blood-brain barrier integrity [2,54]. Activation of the Nrf-2/HO-1 pathway further reduces reactive oxygen species (ROS), aiding recovery [2,54]. PDE5 inhibitors, through PKG activation, protect against mitochondrial dysfunction and promote neuronal survival by activating the PI3K/Akt/mTOR pathway [28].

In traumatic brain injury (TBI), PDE4 inhibition improves cognitive outcomes by raising cAMP levels and reducing neuroinflammation through decreased microglia activation and inflammatory cytokine production [18,36]. PDE5 inhibitors enhance cerebral blood flow, reduce neuronal death, and improve perfusion in damaged brain tissue, suggesting that PDE inhibitors could significantly enhance clinical outcomes [18,36].

In Alzheimer’s disease (AD) models, PDE4 inhibitors improve cognitive function and alleviate depression-like behavior by activating the cAMP/CREB/BDNF pathway, promoting neuroprotection and reducing apoptosis [3,9]. PDE5 inhibitors reduce amyloid-beta (Aβ) and phosphorylated tau levels, prevent neuronal death, and support mitochondrial integrity via the cGMP/PKG/CREB signaling pathway [3,9]. They also promote Aβ clearance and improve vascular function, making PDE5 a promising target for AD therapy [3,9].

## 5. Conclusions

PDE4 and PDE5 inhibitors show great promise as therapeutic agents for neurological disorders like epilepsy, ischemia, TBI, and Alzheimer’s disease. By modulating cAMP and cGMP signaling pathways, they enhance neuroprotection, reduce inflammation, and improve cognitive and vascular functions. While preclinical and clinical studies are encouraging, further research is needed to confirm their long-term efficacy and safety, potentially advancing treatment options for these conditions.

## Figures and Tables

**Table 1:** Observed effects of PDE4 inhibitors in neurological disorders.

PDE4 Inhibitor	Neurological Disorder	Animal Model	Observed Effects	References
Amlexanox	Seizures and Epilepsy	Sprague Dawley rats (6–8 weeks old)	▼ Neuronal cell death ▲ Lysosomal function ▲ Autophagy ▼ Neuroinflammation ▼ Reactive oxygen species (ROS)	[26]
Rolipram FCPR03	Ischemia and Stroke	Adult male C57BL/6 mice (6–8 weeks old) Sprague Dawley rats (6–8 weeks old)	▼ Neuronal cell death ▼ Neuroinflammation ▲ Blood-brain barrier integrity ▼ ROS production ▲SIRT1 and p-AMPK ▲AKT/GSK3β/β-catenin	[2,54]
A33 Rolipram	Traumatic Brain Injury (TBI)	Sprague Dawley rats (6–8 weeks old)	▼ Neuronal cell death ▲ Cognitive function ▲ Synaptic function ▲ Synaptic plasticity ▼ Neuroinflammation	[18,36]
Roflumilast	Alzheimer’s Disease (AD)	APP/PS1 double transgenic mice	▼ Neuronal cell death ▲ cAMP/CREB/BDNF signaling ▲ Bcl-2/Bax ratio ▲ Cognitive function ▼ Depressive-like behavior	[9]

**Table 2:** Observed effects of PDE5 inhibitors in neurological disorders.

PDE5 Inhibitor	Neurological Disorder	Animal Model	Observed Effects	References
Sildenafil	Seizures and Epilepsy	Male Swiss mice Wistar rats	▲ Neuroprotection via NO-cGMP-PKG pathway ▼ ROS production ▼ Neuronal excitability	[34,35]
Sildenafil	Ischemia and Stroke	10-day-old (P10) male Long–Evans rat	▼ Neuronal death ▲ Neuroprotection via PKG pathway ▼ ROS production ▲ PI3K/Akt/mTOR pathway activation ▲ Mitochondrial function	[28]
Sildenafil	Traumatic Brain Injury (TBI)	Twenty-eight Wistar-derived albino strain female rats	▼ Neuronal death ▲ Cerebral blood flow without increasing intracranial pressure ▼ Endothelin-1 levels ▲ Cerebral perfusion	[37]
Mirodenafil	Alzheimer’s Disease (AD)	APP-C105 AD mouse	▼ Amyloid-beta (Aβ) and tau levels ▲ Cognitive function ▲ Aβ clearance via autophagy-lysosome pathway ▲ cGMP/PKG/CREB signaling pathway ▲ Mitochondrial function	[3]

**Table 3:** Focused roadmap for advancing PDE inhibitor research in neurological disorders.

Focus Area	Key Objectives	Specific Actions	Expected Outcomes
Mechanisms of Action	**1.1 Disease-Specific Pathways:** Study PDE4 and PDE5 roles in Alzheimer’s, TBI, stroke, and epilepsy. **1.2 Crosstalk Analysis:** Investigate interactions between PDE4 and PDE5 in overlapping conditions.	- Use single-cell RNA sequencing to identify PDE expression patterns. - Perform proteomic studies in disease-specific animal models. - Analyze effects of selective PDE inhibition in vitro and in vivo.	- Clear understanding of PDE pathways for specific diseases. - Identification of potential targets for dual PDE modulation.
Enhancing PDE Inhibitors	**2.1 Improve Selectivity:** Design isoform-specific inhibitors (e.g., PDE4D, PDE5A). **2.2 Improve Bioavailability:** Ensure BBB penetration and target engagement. **2.3 Safety Testing:** Examine chronic dosing impacts (e.g., emesis, cardiovascular side effects).	- Conduct structure-activity relationship (SAR) studies to refine drug candidates. - Test nanoparticles for enhanced delivery. - Perform 6–12 month chronic toxicity studies in rodents.	- Highly selective inhibitors with reduced off-target effects. - Drugs optimized for safety and efficacy in neurological conditions.
Preclinical to Clinical	**3.1 Validate in Human Models:** Use iPSC-derived neurons and 3D brain organoids to test PDE inhibitors. **3.2 Initiate Clinical Trials:** Prioritize stroke and epilepsy for Phase 1 studies.	- Generate organoids to mimic Alzheimer’s and ischemia environments. - Develop protocols for multicenter trials focusing on safety and efficacy in humans.	- Accelerated clinical translation of PDE inhibitors. - Identification of optimal dosing regimens and therapeutic windows.
Combination Therapies	**4.1 Dual PDE Inhibition:** Explore combined PDE4 and PDE5 targeting. **4.2 Synergistic Approaches:** Pair PDE inhibitors with anti-inflammatory or antioxidant agents.	- Conduct combinatory drug testing in animal models of TBI and epilepsy. - Perform synergy analysis using isobologram techniques.	- Enhanced efficacy through synergistic mechanisms. - Broader therapeutic applications for complex neurological disorders.

## Data Availability

Not applicable.
